# Reviewer Acknowledgement 2013

**DOI:** 10.1186/1472-6823-14-15

**Published:** 2014-03-10

**Authors:** Tim Shipley

**Affiliations:** 1 BioMed Central, Floor 6, 236 Gray's Inn Road, London, WC1X 8HB, UK

## Abstract

**Contributing reviewers:**

The editors of BMC Endocrine Disorders would like to thank all our reviewers who have contributed to the journal in Volume 13 (2013).

## 

Tamuno Alfred

United Kingdom

Alessandro Antonelli

Italy

Marialuisa Appetecchia

Italy

Terence Babwah

Trinidad and Tobago

Christina Bamia

Greece

Arun Bandyopadhyay

India

Michelangela Barbieri

Italy

Abdul Basit

Pakistan

Barbara Batetta

Italy

Lucy Ann Behan

Canada

Albertus Beishuizen

Netherlands

Vassiliki Benetou

Greece

Marian Benroubi

Greece

Elizabeth Beverly

United States of America

Emmanuel Biver

Switzerland

Ulla Bjerre-Christensen

Denmark

Nansi Boghossian

United States of America

Barbara J Boucher

United Kingdom

Maarten Brom

Netherlands

Christa Buechler

Germany

Helena Canhao

Portugal

Claudia Cardoso

Brazil

Federica Carli

Italy

Elisabetta Caselli

Italy

Frederic Castinetti

France

Filomena Cetani

Italy

Chia-Hsuin Chang

Taiwan

Philippe Chanson

France

Ching-Chu Chen

Taiwan

Yanfang Chen

United States of America

Zheng Chen

United States of America

Emanuel Christ

Switzerland

Bjorgulf Claussen

Norway

Aldo Clerico

Italy

Susan Cornell

United States of America

Maria Lucia Correa-Giannella

Brazil

George Coura-Filho

Brazil

Rachel Crowley

Ireland

Michael Croxson

New Zealand

Alberto da Silva Moraes

Brazil

Patric Delhanty

Netherlands

Rachel Desailloud

France

Bruno Detournay

France

Alan Ducatman

United States of America

Anna Egan

United States of America

Joel Ehrenkranz

United States of America

Myrto Eliades

United States of America

Samer El-Kaissi

Saudi Arabia

Sebhat Erqou

United States of America

Vicente Estrada

Spain

Parvis Farahmand

Germany

Andre Faria

Brazil

Asher Fawwad

Pakistan

Wojciech Fendler

Poland

Anna Foryst-Ludwig

Germany

Satoshi Fujii

Japan

Michiaki Fukui

Japan

Martha Funnell

United States of America

Carol Garrison

United States of America

Franz Geisthövel

Germany

Erica Gentilin

Italy

Nigel Glynn

Ireland

Rajna Golubic

United Kingdom

Linda Gonder-Frederick

United States of America

Stefano Gonnelli

Italy

Ravinder Goswami

India

Evanthia Gouveri

Greece

Betul Hatipoglu

United States of America

Zhiheng He

United States of America

Danielle Hessler

United States of America

Eliotte Hirshberg

United States of America

Michael Holick

United States of America

Barry Hudson

United States of America

Muhammad Zafar Hydrie

Canada

Uichi Ikeda

Japan

Prapaporn Jungtrakoon

United States of America

Roman Junik

Poland

Gregory Kaltsas

Greece

Fumihiko Kamezaki

Japan

Katerina Kankova

Czech Republic

Niki Karavitaki

United Kingdom

Darko Kastelan

Croatia

Hillary Keenan

United States of America

Daniel Kelberman

United Kingdom

Karen Knapp

United Kingdom

Meropi Kontogianni

Greece

Bernd Kowall

Germany

Eiji Kutoh

Japan

Tommy Kyaw-Tun

Ireland

Pauline Siew Mei Lai

Malaysia

Vaia Lambadiari

Greece

Lies Langouche

Belgium

Anna Lauber-Biason

Switzerland

Brian Layden

United States of America

Jack Leahy

United States of America

Antonio Lerario

Brazil

Elisabeth Lerchbaum

Austria

Sylvia Ley

United States of America

Stavros Liatis

Greece

Yung-Po Liaw

Taiwan

Urs Lichtenauer

Germany

Unhee Lim

United States of America

Benjamin Longo-Mbenza

South Africa

Nestor Lopez Pompey

Congo

Sten Madsbad

Denmark

Konstantinos Makrilakis

Greece

Rayaz Malik

United Kingdom

Edoardo Mannucci

Italy

Louise Maple-Brown

Australia

Jessica Markowitz

United States of America

Francesco Martino

Italy

Marie McDonnell

United States of America

Pascal Meier

Switzerland

Anderson Melo

Brazil

Alida Melse-Boonstra

Netherlands

Ambrish Mithal

India

Masaki Mogi

Japan

Andrea Montagnani

Italy

Stefano Mora

Italy

José María Moreno-Navarrete

Spain

Kouki Mori

Japan

Brynjulf Mortensen

Denmark

Samiul Mostafa

United Kingdom

Sunni Mumford

United States of America

Tomoaki Murakami

Japan

Satoshi Murao

Japan

Stephen Myers

Australia

Tomoko Nakagami

Japan

Androniki Naska

Greece

Dorit Nitzan Kaluski

Israel

Neil Nokes

United States of America

Bougacha-Elleuch Noura

Tunisia

Jeremy Oats

Australia

Anthonia Ogbera

Nigeria

Helena Oliveira

Brazil

Altan Onat

Turkey

Tríona O'Shea

Ireland

Gopi Krishna Panicker

India

Nikolaos Papanas

Greece

Seung-Jung Park

Korea, South

Renato Pasquali

Italy

Lisa Pastore

United States of America

Carla Pedrosa

Portugal

Michael Piagnerelli

Belgium

Arnoldo Piccardo

Italy

Rosario Pivonello

Italy

Flavia Prodam

Italy

Guillermo Quintas

Spain

Walter Raasch

Germany

Athanasios Raptis

Greece

Dominic Reeds

United States of America

Simon Rees

United Kingdom

Marc Rendell

United States of America

Ulrich Renner

Germany

Han Repping-Wuts

Netherlands

Jordi L Reverter

Spain

Musarrat Riaz

Pakistan

Ana Elisa Rinaldi

Brazil

Ali Rizvi

United States of America

Maria Rosaria rizzo

Italy

Vincenzo Rochira

Italy

Veit Rohde

Germany

Vanessa Ronconi

Italy

José Rosa Neto

Brazil

Marek Ruchala

Poland

Gianni Russo

Italy

Yevgeniy Samyshkin

United Kingdom

Dirk Sander

Germany

Renata Saucedo

Mexico

Jeppe Schroll

Denmark

Albert Selva-O'Callaghan

Spain

Veerasamy Seshiah

India

Amy Shah

United States of America

Abhay Sharma

India

Arundhati Sharma

India

Qiuhua Shen

United States of America

Bingyin Shi

China

Matt Simmonds

United Kingdom

Devada Singh-Franco

United States of America

Fabio Siviero

Brazil

Piotr Skalba

Poland

Brenda Smith

United States of America

Diarmuid Smith

Ireland

Mario Soares

Australia

Stefan Söderberg

Sweden

Soon Song

United Kingdom

Jane Speight

Australia

Stephen Spindler

United States of America

Christopher Stevenson

Australia

Liang Sun

China

Qi Sun

United States of America

Vassiliki Syriou

Greece

Leszek Szewczyk

Poland

Abd Tahrani

United Kingdom

Nicholas Tentolouris

Greece

Stelios Tigas

Greece

Deirdre Tobias

United States of America

Thomas Travison

United States of America

Apostolos Tsapas

Greece

Konstantinos Tsilidis

Greece

Federica Turchi

Italy

Laurie Twells

Canada

Susan van Dieren

Netherlands

Peter van Dijk

Netherlands

Peter M van Hasselt

Netherlands

Andriani Vazeou

Greece

Alejandro Velez Hoyos

Colombia

Maria Cristina Vigone

Italy

Libor Vitek

Czech Republic

Yanwen Wang

Canada

Malgorzata Wasniewska

Italy

Charles Wilkinson

United States of America

Yvonne Winhofer

Austria

Chia-Chao Wu

Taiwan

Min Xu

China

Xilin Yang

China

Yuan Yang

China

Xingwang Ye

China

Baha Zantour

Tunisia

Jiang-Ning Zhou

China

Claudio Zoppi

Brazil

Chao Chun Zou

China

John Zrebiec

United States of America

Aneta Zygulska

Poland

## Pre-publication history

The pre-publication history for this paper can be accessed here:

http://www.biomedcentral.com/1472-6823/14/15/prepub

